# An open, observational, three-arm clinical study of 2–3 cycles of treatment as neoadjuvant therapy in operable locally advanced non-small cell lung cancer: An interim analysis

**DOI:** 10.3389/fimmu.2022.938269

**Published:** 2022-08-19

**Authors:** Linping Gu, Xue Wang, Yile Sun, Yunhua Xu, Xiaomin Niu, Ruiying Zhao, Yaxian Yao, Hong Jian, Yuchen Han, Jinwang Wei, Zhiwei Chen, Shun Lu

**Affiliations:** ^1^ Department of Shanghai Lung Cancer Center, Shanghai Chest Hospital, Shanghai Jiao Tong University, Shanghai, China; ^2^ Department of Pathology, Shanghai Chest Hospital, Shanghai Jiao Tong University, Shanghai, China; ^3^ Department of Data Science, Genomicare Biotechnology (Shanghai) Co., Ltd., Shanghai, China

**Keywords:** operable locally advanced NSCLC, neoadjuvant therapy, major pathological response, tumor regression rate, clinical trial

## Abstract

**Background:**

An open, observational, three-arm clinical study aimed at investigating the efficacy of different neoadjuvant therapies (neoadjuvant immunotherapy with(out) chemotherapy, neoadjuvant chemotherapy, and neoadjuvant targeted therapy) in operable locally advanced non-small cell lung cancer (NSCLC) was conducted (NCT04197076). We report an interim analysis of 49 of 53 evaluable patients.

**Methods:**

This study was conducted at Shanghai Chest Hospital and included eligible NSCLC patients who were 18 years old and had clinical stage IIB–IIIB disease. All 49 patients had surgical resection within 4–6 weeks after 2–3 cycles of neoadjuvant treatment consisting of immunotherapy (24 patients), chemotherapy (16 patients), and a targeted therapy (9 patients) regimen starting on the first day of each 21-day cycle. Pathologic complete response (pCR) was evaluated as the primary endpoint. Major pathological response (MPR) and tumor regression rate (TRR) were also evaluated.

**Results:**

An improved pathologic complete response was achieved in the neoadjuvant immunotherapy arm compared with the neoadjuvant chemotherapy arm and neoadjuvant targeted therapy arm [20.8% (5/24) vs. 6.3% (1/16) vs. 0.0% (0/9); P = 0.089, 95% CI 0.138–0.151]. More importantly, we found that the curative effect of the neoadjuvant immunotherapy arm in pCR+MPR was better than that of the neoadjuvant chemotherapy arm and neoadjuvant targeted therapy arm [45.8% (11/24) vs. 18.8% (3/16) vs. 0.0% (0/9); P = 0.006, 95% confidence interval, 0.008–0.012]. Different neoadjuvant therapies had a statistically significant effect on postoperative pathological tumor downstaging (*P* = 0.017).

**Conclusions:**

Neoadjuvant immunotherapy was associated with a trend toward better pCR than the neoadjuvant chemotherapy arm and neoadjuvant targeted therapy. Curative effect (pCR + MPR) was significantly better with neoadjuvant immunotherapy (P = 0.006, 95% confidence interval, 0.008–0.012).

**Clinical Trial Registration:**

https://clinicaltrials.gov/ct2/show/NCT04197076?recrs=a&cond=NCT04197076&draw=2&rank=1.

## Introduction

Lung cancer is still the leading cause of cancer death in 2020 worldwide. Of lung cancer cases, approximately 80% are classified as non-small cell lung cancer (NSCLC), and approximately one-third of NSCLC cases are diagnosed at a locally advanced stage (1, 2). For locally advanced NSCLC, the standard treatment is multidisciplinary therapy, including neoadjuvant therapy, complete surgical resection, and adjuvant treatment (3, 4). Although neoadjuvant chemotherapy represents a promising treatment strategy that significantly improves the survival rate in operable locally advanced NSCLC, the 5-year survival rate remains less than 50% in these patients (5, 6). Based on this unsatisfactory benefit, researchers have focused on exploring various neoadjuvant therapies in operable locally advanced NSCLC.

The emergence of immune checkpoint inhibitors (ICIs), including programmed cell death 1 (PD-1) or programmed cell death ligand 1 (PD-L1) antibodies and cytotoxic T-lymphocyte-associated protein 4 (CTLA-4) antibodies, has completely revolutionized the situation of neoadjuvant chemotherapy for operable locally advanced NSCLC. Checkmate 159 was the first clinical trial to report neoadjuvant immunotherapy before surgery in 21 limited-stage NSCLC patients who received 2 cycles of nivolumab (7). Then, the NADIM trial (neoadjuvant chemotherapy plus nivolumab) and NEOSTAR trial (neoadjuvant nivolumab or nivolumab plus ipilimumab) also demonstrated the potential value of neoadjuvant immunotherapy in operable NSCLC (8, 9). In addition to neoadjuvant immunotherapy, the EMERGING-CTONG 1103 trial (erlotinib vs. gemcitabine plus cisplatin (GC chemotherapy) as neoadjuvant therapy) also achieved better results in patients with locally advanced epidermal growth factor receptor (EGFR) mutation-positive NSCLC (10). We designed this clinical trial aiming to investigate the efficacy of different neoadjuvant therapies (neoadjuvant immunotherapy with(out) chemotherapy, neoadjuvant chemotherapy, and neoadjuvant targeted therapy) in operable locally advanced NSCLC. Hence, neoadjuvant immunotherapy or neoadjuvant targeted treatment seems to be a promising treatment for operable locally advanced NSCLC. The CheckMate 816 trial showed a significantly longer event-free survival and a higher percentage of patients with a pathological complete response (pCR) than chemotherapy alone in patients with resectable NSCLC (11). In view of the outcome of CheckMate 816, we carried out an interim analysis of the study to evaluate the efficacy of neoadjuvant therapy on pCR, MPR, and TRR before surgery. Overall survival (OS) and progression-free survival (PFS) directly represent patient survival and have been considered the gold standard for evaluating efficacy in many large clinical trials. Pathological complete response (pCR) and major response (MPR) are considered surrogate for PFS and OS (6, 12). Therefore, some clinical trials use pathologic evaluation after neoadjuvant therapy as the endpoint. At present, many studies have shown that a 10% residual viable tumor after surgery in locally advanced NSCLC patients indicates a major pathological response (MPR), and it is ideal for predicting the improvement of long-term prognosis (6, 7, 13, 14). In this context, we report the first clinical results of an interim analysis of the pCR, MPR after surgery, and tumor regression rate (TRR) before surgery in this study after 53 patients were enrolled of which 49 had surgery and are included in analysis for pCR and MPR.

## Methods

### Study design and participants

This open, observational, three-arm clinical study of 2–3 cycles of neoadjuvant targeted therapy, chemotherapy, and immunotherapy with(out) chemotherapy for operable locally advanced NSCLC was conducted at Shanghai Chest Hospital. Biopsy samples of adenocarcinoma were obtained and assessed for EGFR mutation, anaplastic lymphoma kinase (ALK) translocation, and robot operating system (ROS-1) fusion. Patients diagnosed with adenocarcinoma with EGFR mutation, ALK translocation, or ROS-1 rearrangement were offered an appropriate tyrosine kinase inhibitor. The other patients for whom these three mutant genes were not detected were assigned to the immunotherapy ± chemotherapy arm or chemotherapy-alone arm as per discretion of the treating physician. Assessment of PD-L1 expression (22C3 pharmDx kit) was optional.

Eligible patients had Eastern Cooperative Oncology Group performance status 0 or 1, were aged 18 years or older, had cytology or histology documented, had tumor samples available for gene detection (EGFR/ALK/ROS-1), and did not receive antitumor treatment for NSCLC stages IIB–IIIB (American Joint Committee on Cancer 8th edition criteria) that was considered to be surgically operable within 4–6 weeks as assessed by multiple disciplinary teams after 2–3 cycles of neoadjuvant therapy. Patients were excluded from enrollment if they had a history of autoimmune disease, had a malignancy within the past 5 years, or were receiving ongoing treatment with systemic immunosuppressive medications. This study was registered at ClinicalTrials.gov [NCT04197076]. Full inclusion and exclusion criteria are included in the trial protocol. The trial protocol was approved by the institutional review board, and the trial was performed according to the International Conference on Harmonization Good Clinical Practice guidelines. All patients signed informed consent to participate according to the Declaration of Helsinki.

### Procedures

The targeted drugs used against the EGFR 19del/21L858R mutation included afatinib, erlotinib, or gefitinib. Crizotinib was used for EML4-ALK translocation or ROS-1 rearrangement. Different chemotherapy regimens were adopted according to the clinical characteristics following National Comprehensive Cancer Network (NCCN) guidelines and Chinese Society of Clinical Oncology (CSCO) guidelines. Patients in the Immunotherapy group received immunotherapy ± chemotherapy or dual checkpoint inhibitors. Immunotherapy was selected from nivolumab, pembrolizumab, sintilimab, and ipilimumab.

All patients were reviewed for response to therapy at the end of 2–3 cycles (approximately 42 days) according to response evaluation criteria in solid tumors (RECIST) 1.1, and the operation was performed within 4–6 weeks. Necessary radiotherapy, chemotherapy, immunotherapy, or targeted therapy were administered according to NCCN and CSCO guidelines after the operation, and adverse events were evaluated according to common terminology criteria for adverse events (CTCAE) v4.0. The primary endpoint was pCR, which was defined as the lack of all signs of cancer in tissue samples removed during surgery after treatment. The other prespecified outcome was MPR, defined as <10% residual viable tumor, and TRR after neoadjuvant therapy. Patients had the right to withdraw from the trial for any reason at any time. Researchers had the right to withdraw patients from the study due to intolerant toxicity, violation of protocol violation, or other reasons.

After completion of the treatment, patients are being followed up every 3 months for the first 2–3 years, every 4–6 months for an additional 2 years, and annually thereafter. The follow-up evaluations consisted of a physical examination, complete blood count, blood biochemistry, tumor marker, thoracic computed tomography (CT) scan, abdomen B-ultrasound examination, and enhanced CT or magnetic resonance imaging examination of suspected lesions.

### Statistical analysis

The full analysis set, which included all the patients, was used for demographic summaries and efficacy analyses. Continuous variables are expressed as the mean ± standard deviation. Treatment-related adverse events (TRAEs) were defined as adverse events (AEs) with possible or likely attribution to study drugs. All statistical analyses were performed using SPSS (IBM SPSS Statistics 25) and R V.4.1.1, and a two-sided *P* value of less than 0.05 indicated a statistically significant difference.

## Results

### Patient characteristics

From September 2018 to May 2021, 53 operable locally advanced NSCLC patients were enrolled and received 2–3 cycles of neoadjuvant therapy. Twenty-five patients received neoadjuvant immunotherapy (21 patients received immunotherapy + chemotherapy [one patient was from CheckMate-816], one patient received pembrolizumab alone, one patient received sintilimab alone, and two patients received nivolumab + ipilimumab from CheckMate-816), nine patients received neoadjuvant targeted therapy (six patients had EGFR 19del**/**21L858R mutations, two patients had EML4-ALK translocations, and one patient had ROS-1 rearrangements), and 16 patients received neoadjuvant chemotherapy (one patient was from CheckMate-816). **Table 1** presents the characteristics of all patients at baseline.

**Table 1:** Baseline characteristics of all patients (N = 53).

Characteristics	Chemotherapy	Immunotherapy	Targeted therapy
	N = 19 (%)	N = 25 (%)	N = 9 (%)
Age, (years)			
Median (range)	64 (54-77)	60 (43-72)	47 (35-64)
BMI (kg/m2)			
Median (range)	24.2 (19.8-27.0)	25.0 (18.0-27.7)	22.7 (20.8-26.0)
Gender			
Male	16 (84.2)	23 (92.0)	0 (0.0)
Female	3 (15.8)	2 (8.0)	9 (100)
ECOG			
0	2 (10.5)	1 (4.0)	1 (11.1)
1	17 (89.5)	24 (96.0)	8 (88.9)
Smoke			
Never	4 (21.1)	9 (36.0)	9 (100)
Yes/ever	15 (78.9)	16 (64.0)	0 (0.0)
Pathology			
ADC	6 (31.6)	5 (20.0)	8 (88.9)
SCC	12 (63.2)	18 (72.0)	1 (11.1)
Not specified	1 (5.3)	2 (8.0)	0 (0.0)
cT-TNM8			
T1b	1 (5.3)	0 (0.0)	0 (0.0)
T1c	1 (5.3)	1 (4.0)	0 (0.0)
T2a	3 (15.8)	6 (24.0)	7 (77.8)
T2b	5 (26.3)	3 (12.0)	0 (0.0)
T3	4 (21.1)	10 (40.0)	2 (22.2)
T4	5 (26.3)	5 (20.0)	0 (0.0)
cN-TNM8			
N0	3 (15.8)	1 (4.0)	0 (0.0)
N1	3 (15.8)	8 (32.0)	1 (11.1)
N2	13 (68.4)	16 (64.0)	8 (88.9)
TNM8			
IIB	2 (10.5)	3 (12.0)	0 (0.0)
IIIA	10 (52.6)	14 (56.0)	8 (88.9)
IIIB	7 (36.8)	8 (32.0)	1 (11.1)
Gene status			
Wild-type	14 (73.7)	19 (76.0)	0 (0.0)
EGFR mutation	1 (5.3)	0 (0.0)	6 (66.7)
ALK translocation	1 (5.3)	0 (0.0)	2 (22.2)
ROS-1 rearrangement	0 (0.0)	0 (0.0)	1 (11.1)
Unknown	3 (15.8)	6 (24.0)	0 (0.0)
PD-L1 expression			
<1%	2 (10.5)	8 (32.0)	0 (0.0)
1%–50%	3 (15.8)	6 (24.0)	1 (11.1)
>50%	2 (10.5)	7 (28.0)	0 (0.0)
Unknown	12 (63.2)	4 (16.0)	8 (88.9)

BMI, body mass index; ECOG, Eastern Cooperative Oncology Group; ADC, adenocarcinoma; SCC, squamous cell carcinoma; SD, standard deviation; PD, progressive disease; cT-TNM8, clinical T stage according TNM eighth edition; cN-TNM8, clinical N stage according TNM eighth edition; TNM8, stage according TNM eighth edition. TRR, tumor regression rate.

### TRR after 2–3 cycles of neoadjuvant therapy

**Figure 1:**
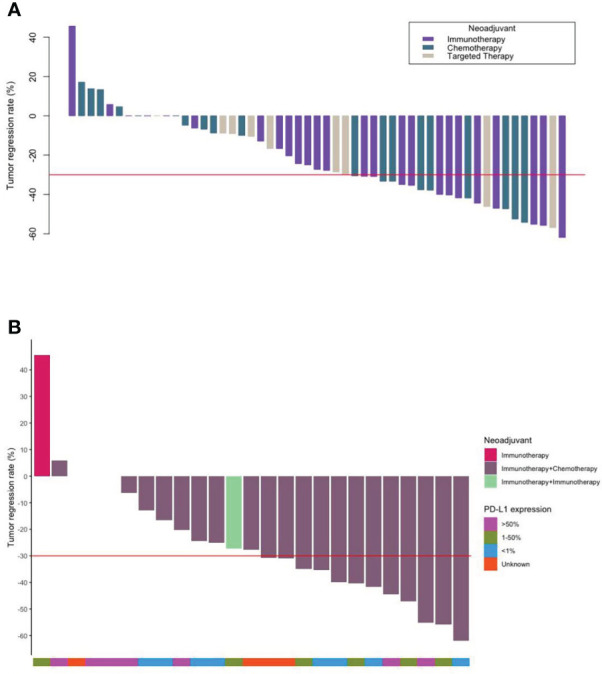
Waterfall plots. **(A)** The tumor regression and number of each type of all patients who underwent neoadjuvant therapy (N = 53). **(B)** The tumor regression of the patients who underwent neoadjuvant immunotherapy and their PD-L1 expression (N = 25).

**Figure 2:**
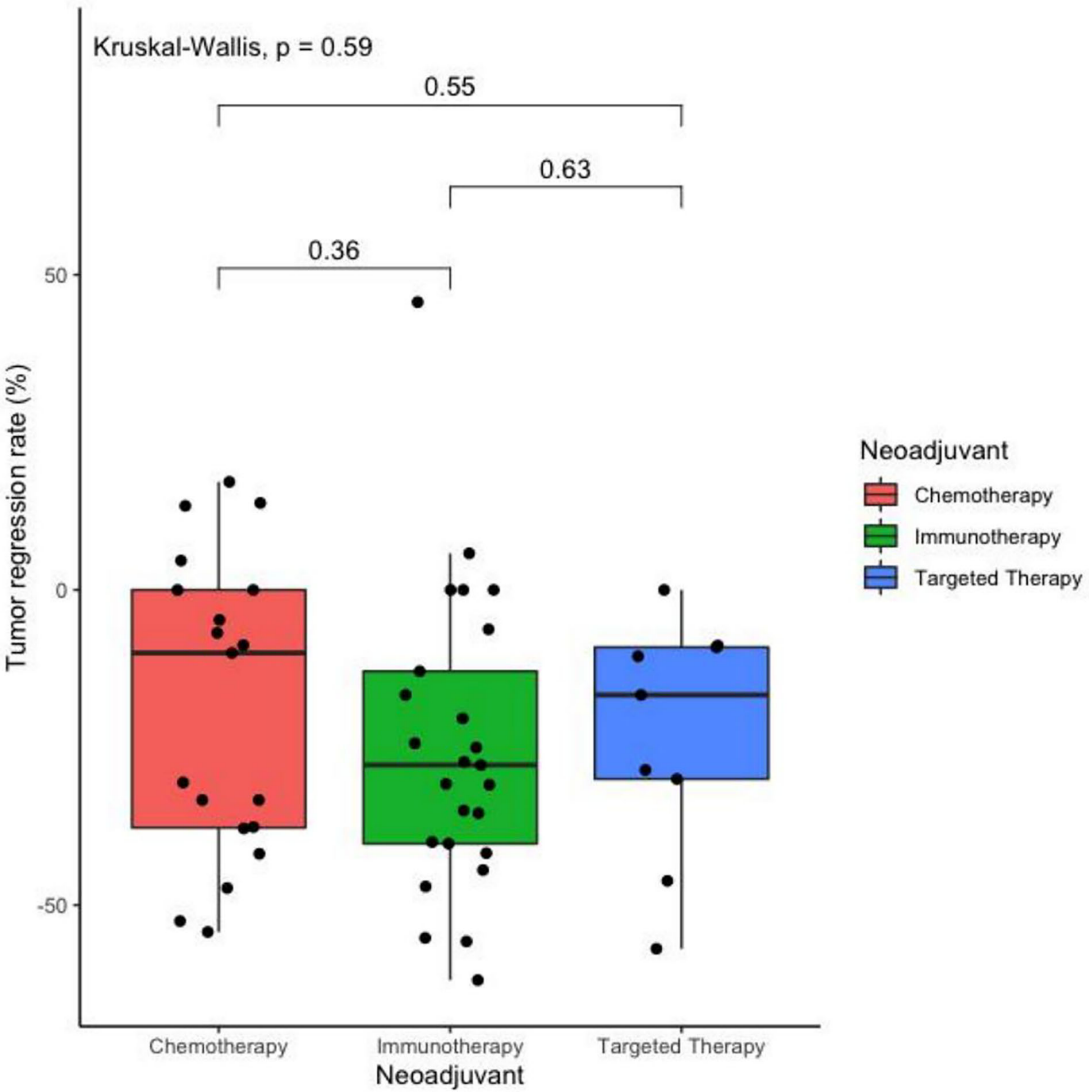
Boxplot of the tumor regression rate after two cycles of different treatments as neoadjuvant therapy in operatable locally advanced NSCLC. (N = 53).

After 2–3 cycles of neoadjuvant therapy, 13 patients (52.0%) achieved PR in the neoadjuvant immunotherapy arm, nine patients (47.4%) achieved PR in the neoadjuvant chemotherapy arm, and four patients (44.4%) achieved PR in the neoadjuvant targeted therapy arm. The TRRs of all patients are shown in **Figure 1A**. No significant differences among the arms were noted (*P* = 0.59) (**Table 2**, **Figure 2**).

**Table 2:** Response and TRR of operable locally advanced NSCLC patients in different neoadjuvant therapy groups after neoadjuvant therapy.

	Chemotherapy	Immunotherapy	Targeted therapy
Characteristics.	N = 19 (%)	N = 25 (%)	N = 9 (%)
RECIST 1.1			
PR	9 (47.4)	13 (52.0)	4 (44.4)
SD	9 (47.4)	11 (44.0)	5 (55.6)
PD	1 (5.3)	1 (4.0)	0 (0.0)
TRR (%)			
Mean ± SD	18.42 ± 23.78	25.10 ± 23.73	22.97 ± 18.99
(range)	(-17.14-54.26)	(-45.65-61.90)	(0.00-56.92)

(N = 53).

### Surgery

Forty-nine patients were scheduled to successfully undergo surgery within 4–6 weeks after 2–3 cycles of neoadjuvant therapy. In the neoadjuvant immunotherapy arm, 23 patients (23/24, 95.8%) achieved complete tumor resection (R0), including eight patients (8/9, 88.9%) in the neoadjuvant targeted therapy arm and 15 patients (15/16, 93.8%) in the neoadjuvant chemotherapy arm. **Table 3** shows all the characteristics of the surgical outcomes. Nineteen patients (79.2%) achieved pathological tumor downstaging in the neoadjuvant targeted therapy arm compared with four patients (44.4%) in the neoadjuvant targeted therapy arm and six patients (37.5%) in the neoadjuvant chemotherapy arm. Additionally, a significant difference in pathological tumor downstaging was noted among the different neoadjuvant therapy arms (*P* = 0.017).

**Table 3:** Tumor location, surgical approach, and surgical outcomes of operable locally advanced NSCLC patients in different neoadjuvant therapy groups.

	Chemotherapy	Immunotherapy	Targeted therapy	
Characteristics.	N = 16 (%)	N = 24 (%)	N = 9 (%)	P
Tumor location				0.866^a^
LUL	4 (25.0)	7 (29.2)	2 (22.2)	
LLL	1 (6.3)	3 (12.5)	1 (11.1)	
RUL	6 (37.5)	10 (41.7)	2 (22.2)	
RML	2 (12.5)	1 (4.2)	2 (22.2)	
RLL	3 (18.8)	3 (12.5)	2 (22.2)	
Approach				0.065^a^
VATS	4 (25.0)	6 (25.0)	6 (66.7)	
Thoracotomy	12 (75.0)	18 (75.0)	3 (33.3)	
Surgical margin				0.780^a^
R0	15 (93.8)	23 (95.8)	8 (88.9)	
R1	1 (6.1)	1 (4.2)	1 (11.1)	
ypT-TNM8				0.022^a^
T0	1 (6.3)	5 (20.8)	0 (0.0)	
T1b	0 (0.0)	1 (4.2)	2 (22.2)	
T1c	1 (6.3)	8 (33.3)	1 (11.1)	
T2a	6 (37.5)	4 (16.7)	4 (44.4)	
T2b	0 (0.0)	2 (8.3)	0 (0.0)	
T3	6 (37.5)	2 (8.3)	2 (22.2)	
T4	2 (12.5)	2 (8.3)	0 (0.0)	
ypN-TNM8				0.405^a^
N0	6 (37.5)	11 (45.8)	4 (44.4)	
N1	3 (18.8)	5 (20.8)	0 (0.0)	
N2	7 (43.8)	7 (29.2)	5 (55.6)	
N3	0 (0.0)	1 (4.2) ^b^	0 (0.0)	
yp-TNM8				0.147^a^
Stage 0	1 (6.3)	5 (20.8)	0 (0.0)	
IA	0 (0.0)	3 (12.5)	1 (11.1)	
IB	2 (12.5)	2 (8.3)	1 (11.1)	
IIB	2 (12.5)	6 (25.0)	2 (22.2)	
IIIA	9 (56.3)	4 (16.7)	5 (55.6)	
IIIB	2 (12.5)	3 (12.5)	0 (0.0)	
IVA	0 (0.0)	1 (4.2)	0 (0.0)	
Pathological downstaging				
T stage				0.017^a^
Yes	6 (37.5)	19 (79.2)	4 (44.4)	
No	10 (62.5)	5 (20.8)	5 (55.6)	
N stage				0.956^a^
Yes	8 (50.0)	12 (50.0)	4 (44.4)	
No	8 (50.0)	12 (50.0)	5 (55.6)	
TNM stage				0.986^a^
Yes	9 (56.3)	14 (58.3)	5 (55.6)	
No	7 (43.8)	10 (41.7)	4 (44.4)	

(N = 49)*. LUL, left upper lobe; LLL, left lower lobe; RUL, right upper lobe; RML, right middle lobe; RLL, right lower lobe; VATS, video-assisted thoracic surgery; ypT-TNM8, ypT stage according TNM eighth edition; ypN-TNM8, ypN stage according TNM eighth edition;

a likelihood ratio.

b this patient was pathologically evaluated as N3 after surgical treatment due to the lymph nodes of the contralateral 4R group and 10R group were obtained, and the pathological diagnosis was positive.

*, 49 patients (49/53, 92.5%) had underwent surgical resection with curative intent. Four patients did not undergo surgery after neoadjuvant therapy. Three patients were in the chemotherapy arm, and one patient with squamous cell carcinoma was in the immunotherapy arm. In the chemotherapy arm, one patient with adenocarcinoma had refused surgical resection after neoadjuvant chemotherapy. Although the efficacies of two patients with squamous cell carcinoma in the chemotherapy arm were evaluated as SD after neoadjuvant chemotherapy, the lesions were enlarged, and the investigators evaluated that those two patients did not have the possibility of complete surgical resection, so they did not receive surgical resection. Later, these two patients received local radiotherapy. One patient in the immunotherapy group did not receive surgical treatment because of the failure of neoadjuvant therapy for disease progression.

### Pathological response rate

**Figure 3:**
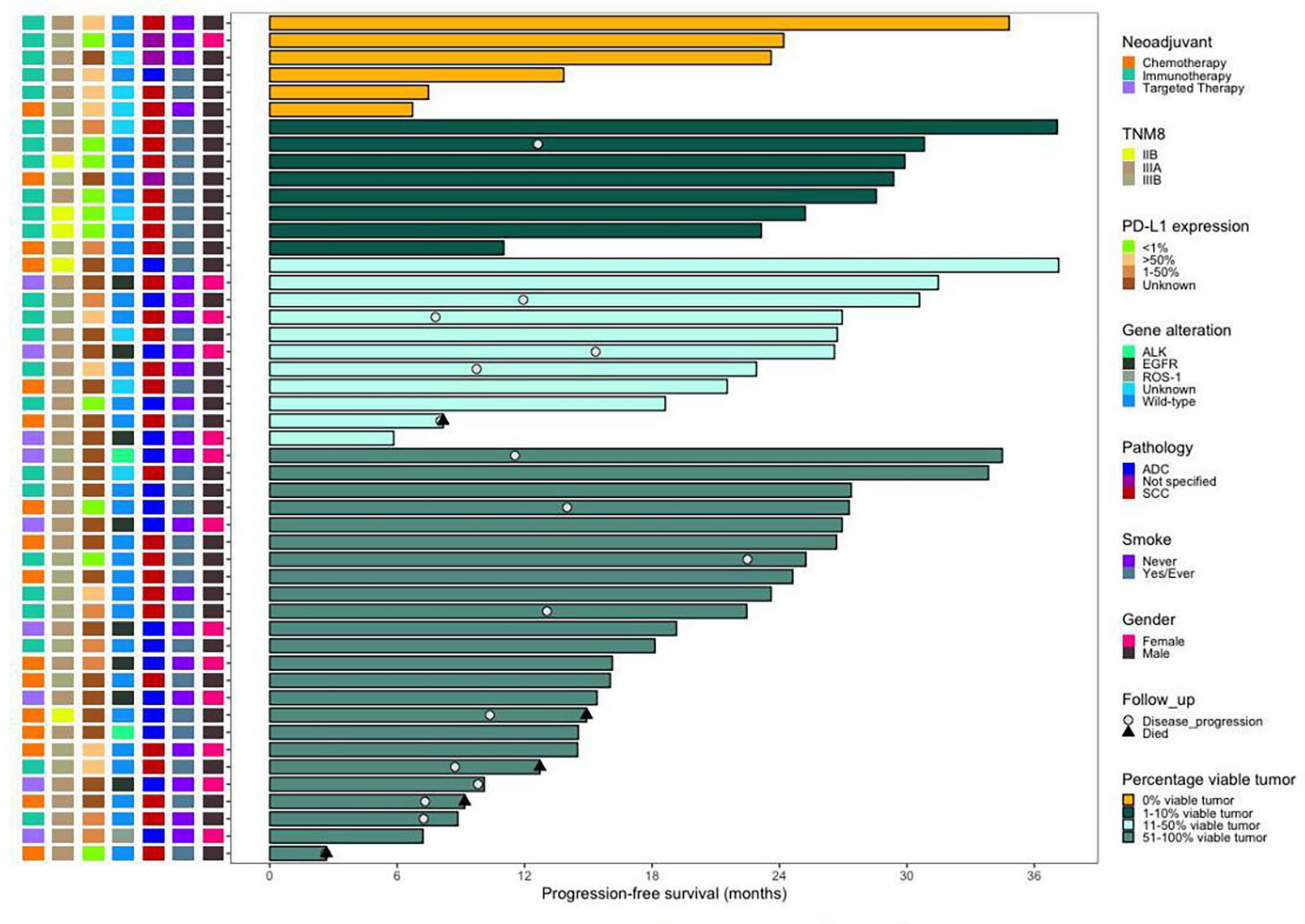
Swimming plot of progression-free survival in the patients who underwent surgery. (N = 49). Each bar represents one patient. The left column shows clinical characterestics. Date cutoff was Nov. 4, 2021, sixteen (32.7%) patients who underwent surgery had disease progression, five (10.2%) of whom died. Of the 14 patients with pCR or MPR, one patient who received neoadjuvant immunotheraphy had disease progression. Of the 16 (one patient has died), 5 patient received neoadjuvant immunotheraphy (four patients have died), and 3 patients received neojuvant targeted chemotheraphy.

The characteristics of 49 patients in different percentage viable tumor groups are shown in **Figure 3**. Five patients (20.8%) presented pCR in the neoadjuvant immunotherapy arm, and one patient (6.3%) achieved pCR in the neoadjuvant chemotherapy arm. However, no patients presented pCR in the target arm. Moreover, eight patients presented with MPR, including six patients (25%) in the neoadjuvant immunotherapy arm and two patients (12.5%) in the neoadjuvant chemotherapy arm. pCR+MPR significantly differed among the different neoadjuvant therapy arms (*P* = 0.006; 95% confidence interval [CI], 0.008–0.012) (**Table 4**).

**Table 4:** Pathological response rates of operable locally advanced NSCLC patients in different neoadjuvant therapy groups after surgery.

Percentage viable tumor	Total	Chemotherapy	Immunotherapy	Targeted therapy	
	N = 49 (%)	N = 16 (%)	N = 24 (%)	N = 9 (%)	P
pCR +MPR	14 (28.6)	3 (18.8)	11 (45.8)	0 (0.0)	0.006(0.008-0.012)
pCR(0% viable tumor)**^a^**	6 (12.2)	1 (6.3)	5 (20.8)	0 (0.0)	0.089(0.138-0.151)
MPR(1%–10% viable tumor)**^b^**	8 (16.3)	2 (12.5)	6 (25.0)	0 (0.0)	
11%–50% viable tumor	11 (22.4)	3 (18.8)	5 (20.8)	3 (33.3)	
51%–100% viable tumor	24 (49.0)	10 (62.5)	8 (33.3)	6 (66.7)	

(N = 49).

### Adverse events and long-term follow-up

The toxicity of the neoadjuvant immunotherapy arm was manageable overall, and no new safety concerns, including operative mortality, were noted. By 4 November 2021, 16 patients (five patients in the neoadjuvant chemotherapy arm, eight patients in the neoadjuvant immunotherapy arm, and three patients in the neoadjuvant targeted arm) had progressive disease, and five of these patients (four patients in the neoadjuvant chemotherapy arm and one patient in the neoadjuvant immunotherapy arm) died (**Figure 3**).

### Subgroup analysis

In the subgroup analysis, TRR and pCR+MPR were observed regardless of PD-L1 expression (*P* = 0.859, 0.053), and PD-L1 expression did not seem to predict the benefit of neoadjuvant benefit (**Figure 1B**, **Table 5**). Neoadjuvant immunotherapy was better than neoadjuvant chemotherapy in terms of TRR (30.76 ± 18.13 vs. 23.57 ± 22.16) and pCR+MPR (47.6% vs. 18.8%), but the difference was not significant (*P* = 0.399, 0.063) (**Table 5**).

**Table 5:** The results in different groups of PD-L1 expression and treatments.

	Expression of PD-L1^a^			Chemotherapy	Immunotherapy + chemotherapy	
Characteristic	<1%, N = 8 (%)	≥1%, N = 10 (%)	P	N = 16 (%)	N = 21 (%)	P
TRR (%)			0.859^b^			0.399^b^
Mean ± SD(range)	32.25 ± 15.91(12.90-61.90)	31.12 ± 21.89(-5.80-55.77)		23.57 ± 22.16(-17.14-54.26)	30.76 ± 18.13(-5.80-61.90)	
Percentage viable tumor			0.053^c^			0.063^c^
pCR+MPR	6 (75.0)	3 (30.0)		3 (18.8)	10 (47.6)	
Non-pCR/MPR	2 (25.0)	7 (70.0)		13 (81.3)	11 (52.4)	

a all patients who were detected PD-L1 in neoadjuvant immunotherapy + chemotherapy. (N=18).

b Mann–Whitney U test.

c likelihood ratio.

## Discussion

Although the efficacy of available treatment methods for operable locally advanced NSCLC has continuously improved in recent years, most of the relevant clinical trials employ OS and PFS as the primary endpoints, which not only leads to the high cost of time-consuming clinical trials but also hinders research on and the development of newly marketed drugs (12). It seems that the pathologic response could represent an interim surrogate analysis endpoint for OS because pathological assessment, which includes pCR and MPR, can be available using primary lesions resected during surgery in patients undergoing neoadjuvant therapy based on some previous studies (6, 9, 13, 15, 16). Neoadjuvant immunotherapy has a better clinical effect than neoadjuvant chemotherapy and neoadjuvant-targeted therapy based on pCR, MPR, TRR, or pathological T downstaging. The toxicity of neoadjuvant immunotherapy was generally controllable, and no new safety problems, including the operation mortality rate, were noted. The efficacy of neoadjuvant immunotherapy was not related to PD-L1 expression in subgroup analysis.

A previous review reported that the median rate of pCR from 15 clinical trials of neoadjuvant chemotherapy was 4% (range 0%–16%) (6). Mouillet et al. also retrospectively analyzed 492 patients with stage IB or II NSCLC who received 2 cycles of neoadjuvant chemotherapy. In total, 41 patients (8.3%) achieved pCR, and it was a favorable prognostic factor for OS in multivariate analysis (RR = 0.34; 95% CI = 0.18–0.64) (17). Compared with previous studies, only one patient (1/16, 6.3%) in the neoadjuvant chemotherapy arm achieved pCR in this study. Pataer et al. found that the percentage of viable tumor cells was significantly associated with OS (*P* = 0.005; HR = 1.01; 95% CI = 1.00–1.02), and 19% of patients (39/192) achieved MPR after neoadjuvant chemotherapy in a retrospective study (18). Weissferdt et al. also reported that MPR is related to OS (*P* < 0.01; HR = 2.68) after reevaluating the postoperative pathological specimens of 151 patients with operable NSCLC who received neoadjuvant chemotherapy, and 22% of patients (33/151) achieved MPR (19). Similar to previous studies, three patients (3/16, 18.8%) reached MPR after neoadjuvant chemotherapy in the present study.

For patients with driver gene-positive NSCLC, several studies have reported some positive results regarding neoadjuvant treatment. EMERGING-CTONG 1103 was a multicenter (17 centers in China), open-label, phase II, randomized controlled trial of erlotinib vs. GC chemotherapy as neoadjuvant/adjuvant therapy in patients with stage IIIA-N2 non-small cell lung cancer with EGFR mutations in exon 19 or 21 (10). In the erlotinib group, three patients (3/31, 9.7%) achieved MPR compared to only one patient (1/24, 4.2%) who achieved MPR in the GC chemotherapy group. Another single-arm, phase II trial that aimed to investigate the efficacy and safety of preoperative gefitinib in 35 patients with stage II–IIIA operable NSCLC reported that the rate of MPR was 24.2% (20). Zhang et al. also described 11 ALK receptor tyrosine kinase gene (ALK)-positive patients with pathologically confirmed N2 NSCLC who were treated with neoadjuvant crizotinib and suggested that neoadjuvant crizotinib might be feasible and well tolerated in locally advanced diseases for complete resection (21). However, in the current study, no patients reached pCR or MPR in the neoadjuvant-targeted therapy arm. The most important limitation of this study could be that only nine patients were included in the neoadjuvant-targeted therapy arm, which was far less than the number of patients in the neoadjuvant chemotherapy arm and neoadjuvant immunotherapy arm. However, the effect of TRR and pathological tumor downstaging in the neoadjuvant-targeted therapy arm was better than that noted for neoadjuvant chemotherapy. Hence, more prospective clinical trial data are needed to further support the application of pCR/MPR in neoadjuvant-targeted therapy.

The remarkable effect of immunotherapy in advanced NSCLC brings the dawn for operable NSCLC and prompts researchers to conduct a large number of clinical trials in operable locally advanced NSCLC. Moreover, PD-1/L1 inhibitors combined with platinum-based chemotherapy are widely used in early neoadjuvant clinical trials because neoadjuvant chemotherapy has been demonstrated to enhance PD-L1 expression and promote the infiltration of immune cells in tumors (22). NADIM was the first phase II trial to explore 3 cycles of neoadjuvant immunotherapy + chemotherapy (nivolumab plus paclitaxel–carboplatin regimen) in patients with operable clinical stage IIIA NSCLC, and 41 patients (41/46, 89%) successfully underwent R0 resection (8). Moreover, the rates of MPR and pCR were 83% (34/41) and 63% (26/41), respectively. Another clinical trial of neoadjuvant atezolizumab and paclitaxel–carboplatin in resectable stage IB-IIIA NSCLC also showed that 26 (26/30, 87%) patients underwent successful R0 resection, and the rates of MPR and pCR were 57% and 33%, respectively (13). CheckMate 816 was the first phase III trial to confirm the benefit of nivolumab plus platinum-doublet chemotherapy as neoadjuvant treatment for resectable NSCLC. Neoadjuvant nivolumab and platinum-doublet chemotherapy significantly improved the pCR, MPR, and R0 resection rates compared with neoadjuvant chemotherapy (24% vs. 2.2%; 36.9% vs. 8.9%; 83% vs. 78%), and patients who underwent neoadjuvant nivolumab and platinum-doublet chemotherapy all experienced benefits regardless of disease stage, histology, and PD-L1 expression levels (11, 23). Our interim analysis showed that the patients in the neoadjuvant immunotherapy arm had the best benefits, including MPR (25% vs. 12.5% vs. 0.0%) or pCR (20.8% vs. 6.3% vs. 0.0%), compared with the neoadjuvant chemotherapy arm or neoadjuvant-targeted therapy arm. This finding also verified the value of neoadjuvant immunotherapy in pathological tumor downstaging (79.2% vs. 37.5% vs. 44.4%) and successful R0 resection (95.8% vs. 93.8% vs. 88.9%). Although, in our study, the incidence of pCR was not significantly different in the three groups, we suggest that the small sample size explains why the results did not achieve statistical significance. Similar to our results, another phase II study of 2–4 cycles of neoadjuvant immune chemotherapy for resectable stage IIIA NSCLC reported MPR and pCR values of 43.3% and 20%, respectively (24). One reason could be that for operable NSCLC patients, the use of ICIs as neoadjuvant treatment is beneficial to reactivate the activity of antitumor immune T cells and improve the ability to remove potential micrometastases in the body (25). Another reason could be the presence of more gene mutations in tumor cells that produce new epitopes after chemotherapy, which can enhance the immunogenicity of the tumor and improve the efficacy of immunotherapy (26, 27). Although the results of many studies have suggested a correlation between PD-L1 expression and immunotherapy efficacy, even patients with a negative expression of PD-L1 can still benefit from neoadjuvant immunotherapy in subgroup analysis.

In our study, six patients (12.2%, 6/49) achieved pCR, including four stage IIIA NSCLC patients and two stage IIIB NSCLC patients (**Figure 3**). When evaluating the efficacy of neoadjuvant immunotherapy before the operation, special attention should be given to pseudoprogression and hyperprogression. Surgical treatment after neoadjuvant immunotherapy was well tolerated with no significant delays to surgery or unexpected surgical complications.

Several limitations to this study should be noted. First, this was an interim analysis, and we hope to observe the OS and PFS of this study in the future. Second, we did not analyze the mechanism of different neoadjuvant arms, especially in the neoadjuvant immunotherapy and neoadjuvant-targeted therapy arms. Third, this was a single-center study with a small sample, which limited its applicability to some extent. In the future, we should focus on studying the mechanism of different neoadjuvant therapies and screening the best neoadjuvant therapy mode to prolong the survival rate of operable NSCLC. Whether this pathological response results in prolonged survival requires further analysis in a larger patient cohort.

## Conclusion

In conclusion, neoadjuvant immunotherapy appears to be safe and feasible and does not increase perioperative morbidity following surgery. The existing data suggest that neoadjuvant immunotherapy is promising in terms of pCR, MPR, and TRR among patients with operable locally advanced NSCLC compared with neoadjuvant-targeted therapy and neoadjuvant chemotherapy. Neoadjuvant immunotherapy could therefore represent a potential therapeutic strategy for operable locally advanced NSCLC patients.

## Data availability statement

The original contributions presented in the study are included in the article/supplementary material. Further inquiries can be directed to the corresponding authors.

## Ethics statement

The studies involving human participants were reviewed and approved by the IRB of Shanghai Chest Hospital (No. KS1971). All patients had provided consent for the use of the medical information contained in this trial for research and publication. The patients/participants provided their written informed consent to participate in this study.

## Author contributions

Conceptualization: ZC, XN, and LG; data curation: LG, XW, and YS; formal analysis: LG, XW, and YS; funding acquisition: ZC and XN; investigation: YX, XN, RZ, YH, YY, and HJ; methodology: LG, XW, YS, and ZC; project administration: LG, ZC, and SL; resources: LG; software: LG, XW, and JW; supervision: YX, XN, YH, YY, and HJ; Validation: LG, XW, YS, YX, XN, YH, YY, HJ, and ZC; visualization: XW. All authors read and approved the final manuscript.

## Funding

The Technology Transfer Project of Shanghai Jiao Tong University School of Medicine (No. ZT202010); the National Natural Science Foundation of China (No. 81972187); Projects of the Committee of Shanghai Science and Technology (Nos. 19ZR1449800 and 20Y11913700); Projects of Shanghai Municipal Public Health Bureau (Nos. 201840122 and 2019SY048); Doctoral Innovation Fund of Shanghai Jiao Tong University School of Medicine (No. BXJ201952); Interdisciplinary Program of Shanghai Jiao Tong University (No. YG2017MS80); Project of Shanghai Talent Development Fund (No. 2019074); National Natural Science Foundation of China (82030045); National Multi-disciplinary Treatment Project for Major Diseases (2020NMDTP); Technology Innovation Program of Shanghai (19411950500); and the National Key R&D Program of China (2016YFC1303300).

## Acknowledgments

The authors thank GenomiCare Biotechnology (Shanghai) for their effort in Gene analysis in the neoadjuvant-targeted therapy arm.

## Conflict of interest

Author JW is employed by GenomiCare Biotechnology (Shanghai) Co, Ltd.

The remaining authors declare that the research was conducted in the absence of any commercial or financial relationships that could be construed as a potential conflict of interest.

## Publisher’s note

All claims expressed in this article are solely those of the authors and do not necessarily represent those of their affiliated organizations, or those of the publisher, the editors and the reviewers. Any product that may be evaluated in this article, or claim that may be made by its manufacturer, is not guaranteed or endorsed by the publisher.
